# Clinical training in veterinary medicine: perspectives on quality and the role of specialists

**DOI:** 10.3389/fvets.2025.1601783

**Published:** 2025-06-25

**Authors:** L. Drzemalla, C. Kleinsorgen, A. Tipold

**Affiliations:** ^1^Center for Teaching, University of Veterinary Medicine Hannover, Foundation, Hannover, Germany; ^2^Clinic for Small Animals, University of Veterinary Medicine Hannover, Foundation, Hannover, Germany

**Keywords:** practical year, clinical training, elective practical training, day one competences, specialization

## Abstract

The practical year represents the transition between studies and professional work for students and is therefore a significant phase of clinical undergraduate training in Germany. Veterinary educational establishments are obliged to obtain information on elective practical training in order to assess the quality of clinical training. Since the constant increase in knowledge makes it necessary in many cases for veterinarians to specialize as part of postgraduate training, the influence of these specializations on clinical training of undergraduates should be investigated using a mixed-method design. An online survey was created for students and veterinarians as part of the quantitative data collection. Qualitative data collection was carried out in the form of guided interviews using a mixed-method design, with questions based on preliminary, selected results from the questionnaires. The majority of students surveyed were satisfied with the quality of training and supervision during their practical year (73%). The specialization of veterinarians was rated as “(very) important” by 52% for choosing a work placement for practical training. Around 40% of students felt that they received different levels of supervision from veterinarians with different qualifications. The veterinarians rated theoretical knowledge, communication skills and commitment of the students predominantly positively, but noted a deficit in practical skills. The specialization of veterinarians has an influence on theoretical education within the clinical training. The quality of training generally depends on the veterinarians’ level of knowledge and professional experience. The practical year gives students the opportunity to improve their practical skills before entering the profession, however for better outcomes and increased satisfaction, the objectives for elective practical training should be defined in advance between the practical training provider and the student.

## 1 Introduction

It is the aim of veterinary education to qualify students for their life as scientifically and practically trained veterinarians (1). As part of this training, the practical year represents the transition between studies and the subsequent professional career (2) and is thus significantly involved in the clinical training of students. In Germany, the final year in veterinary education is known as the “practical year” and is divided in veterinary public health activities, training in public veterinary services and training in a veterinary practice or veterinary clinic (1). A clinic typically refers to lager veterinary institution, often with multiple veterinarians and more advanced equipment, such as university or referral clinics and opening hours for 24/7. Practice on the other hand, refers to smaller, often privately owned establishments with generalist scope, opening hours for 24/7 are not necessary. The elective clinical training is performed intramurally in the clinics of the veterinary education establishments (VEE) and extramurally in private practices and clinics (1). Elective practical training is a standardized term (3) used for “*training periods, which are an integral part of the curriculum, and which may be taken either outside the VEE under the supervision of a qualified person* (e.g.*, a practitioner*) *or intra-murally, the student being under the supervision of a teaching staff or a qualified person. They should be available to all students but, like all elective activities, their contents may* var*y from one undergraduate student to another.”* In Germany, students have to complete obligatory practical training comprising a total of 850 extramural hours (1 × 4 weeks and 2 × 2 months) spent in private practice or in a clinic.

Nevertheless, lack of practical training and lack of relevance of some teaching content is repeatedly criticized (4–9). For this reason, a stronger focus on practical-clinical training is called for in order to reduce stress levels, increase motivation and better prepare students to start their careers (5, 6, 9–11). To ensure the quality of veterinary education, the European Association of Establishments for Veterinary Education (EAEVE) monitors the minimum standards for the veterinary degree program laid down in Directive 2005/36/EC of the European Union. Part of these minimum standards are the “Day One Competences” that are expected of students at the time of graduation and for which the veterinary educational establishments are responsible (3). According to the Standard Operating Procedure (SOP) of the European System of Evaluation of Veterinary Training (ESEVT), the educational establishments must be informed about the circumstances of extramural clinical training (3). Checklists and logbooks are used as a means of performance assessment, self-monitoring and skills acquisition (12–14). While students of human medicine already gain practical experience during their clinical clerkship before the practical year (15), students of veterinary medicine have this opportunity in facilities such as the clinical skills lab or during participation in evening treatments or electives and during clinical workplace training (14). The clinical skills lab and training on simulators is an important tool for clinical training, as the improper performance of some activities can be associated with a high risk for the patient (16, 17). Independence and commitment are expected from both veterinary and human medicine students, and are considered important for clinical training (17, 18). A higher standardization of elective clinical training is implemented in Germany through the introduction of uniform training agreements, uniform evaluation questions and didactic training courses offered by establishments for veterinary education in Germany (19). In addition to mandatory continuing education for doctors of human medicine and veterinarians (20, 21), there is the possibility of standardized postgraduate training (22, 23). In Germany, specialization is governed by the regulations of the Chamber of Veterinarians or Medical Chamber. The constant increase in knowledge makes it necessary for more and more veterinarians to specialize in treating single species, specialize according to organ systems or treatment methods (24). In Germany, in addition to the international training to become a European Diplomate (25), there is accredited national specialization (22). The veterinarians can become a veterinary specialist, for example, for small animals or horses, or they can become a veterinarian with additional qualifications in, for example, ophthalmology or cardiology. In this study, the term ´small animals´ refers specifically to dogs and cats, whereas ´small mammals´ describes species such as guinea pigs and rabbits. Apart from the veterinarians, there are also veterinary paraprofessionals included in the supervision of students during their practical training (26). These paraprofessionals include, for example, veterinary nurses or veterinary technicians.

The aim of this study is to explore the quality of supervision and clinical training of students during the practical year and to determine the possible influence of existing specializations of supervising veterinarians on the quality of training. In addition, theoretical, practical and communication skills and attitudes that students already have before the practical year and whether they are able to apply the Day One Competences will be investigated.

## 2 Materials and methods

A mixed-method design was used to collect both quantitative and qualitative data from students and veterinarians (27). The mixed-method design is a suitable research method for studies in medical education and can thus contribute to optimization of teaching (28) by achieving a deeper understanding of the topic (29). As part of an explanatory sequential research design, quantitative data was first collected by means of a survey. Data obtained in this survey could be used to develop the interview guidelines. The topics covered in the surveys were supplemented and deepened by qualitative interviews in which personal experiences were gathered (30). The surveys were created on the basis of previously defined questions. Overall, the topics “Demographics,” “Practical year,” “Self assessment” by students, “Assessment of students” by veterinarians and “Student satisfaction” were surveyed (Supplementary material). To ensure the data set was as comprehensive as possible, it was agreed in advance that only questionnaires that were fully completed or had only single missing values would be included in the analysis. Questionnaires in which entire thematic sections were left unanswered were excluded. Questionnaires with single missing answers in a section on the other hand, were included.

Students at veterinary educational establishments and practicing veterinarians in Germany were surveyed. The students were reached via the semester-related e-mail distribution lists and the bvvd e. V. (Federal Association of Veterinary Medicine Students in Germany). In total, the survey was made available to approximately 2,270 students (31). These were students either at or nearing the end of their practical year. The link to the survey was made available to veterinarians via newsletters from the Lower Saxony Chamber of Veterinarians and the German Veterinary Medical Association (DVG). Volunteers for the interviews were recruited via the social media channels of the University of Veterinary Medicine Hannover. Since certain requirements had to be met by the participants, a purposive sample was carried out. Students should have already completed one period of elective practical training within their practical year. The veterinarians were selected taking into account their affiliation to different institutions (clinic, practice), varying animal species and specializations.

The surveys were conducted using the online software LimeSurvey® (LimeSurvey GmbH, Hamburg, Germany). and were recorded and processed for descriptive and statistical analysis using the program Microsoft® Excel 2023 (Microsoft Corporation, California, USA). Observational statistics are presented in one section of the manuscript to provide an overview of the available data without over interpreting the findings. Free text responses were recorded using qualitative content analysis according to Mayring et al. (32) and Braun and Clarke (33). The interviews were conducted and recorded via Microsoft^®^ Teams. The automatic transcription by Microsoft® Teams was then checked for accuracy and meaningfulness using the video and audio recordings and edited if necessary. To carry out a thematic content analysis according to Braun and Clarke (33), an initial data review was carried out to obtain an overview of the material and to record initial observations. In the next step, sections relevant to the research questions were coded using the MAXQDA program (version 24.7.0) and categories or themes were created as part of an inductive category formation process based on the data and the codes already created, which were then defined in more detail. For the final evaluation, the categories and themes were analyzed and interpreted in relation to the research questions. The AI system DeepL was used in part for the translation (34).

The data collection and analysis is subject to the data protection regulations Art. 6 | 1 lit. e in conjunction with 89 GDPR, §3 | 1 No. 1 NHG (Lower Saxony Higher Education Act) and §13 NDSG (Lower Saxony Data Protection Act) as well as Art. 9 I, II lit. a GDPR. The study design, data policy and consent forms were reviewed and approved by the data protection officer of the University of Veterinary Medicine Hannover.

## 3 Results

### 3.1 Demographics

The online surveys with students and veterinarians were conducted from 8th January 2024 to 10th July 2024. In order to obtain reports from students and veterinarians from different elective practical training areas and periods, the survey was available for more than 6 months. Selected results of this study are described below. Not all questions are discussed in detail.

From students a total of 231 questionnaires were submitted, 132 of these were excluded from the analysis due to incompleteness. Finally, questionnaires of 99 students could be fully evaluated, who assessed a total of 124 practical training periods. The median age of the students was 25 years (minimum 22 years, maximum 47 years). Overall, 92% (*n* = 91) of the students stated that they were female, 7% (*n* = 7) were male and in one questionnaire gender was not specified. Before studying veterinary medicine, 24% (*n* = 24) of the participating students had completed and 13% (*n* = 13) of students had started an apprenticeship or another course of study. Overall, the started and completed courses and training before studying veterinary medicine could be categorized as “(veterinary) medicine,” “natural sciences,” “agriculture” and “other.” The veterinary medicine category includes training as a veterinarian paraprofessional, for example veterinary nurses or veterinary technicians. In total, 53% (*n* = 52) of the participating students came from the University of Veterinary Medicine Hannover. 26% (*n* = 26) of the students came from the University of Gießen, 11% (*n* = 11) from the University of Leipzig and 10% (*n* = 10) from the University of Berlin. No students from the University of Munich took part in the survey.

The interviews were conducted with eight students from various veterinary establishments in Germany between October 2024 and December 2024 and lasted between 6:56 min and 30:29 min. The participants’ age ranged from 23 to 34 years. A total of seven participants were female and one was male. Of the participants, six had not started or completed any apprenticeship or studies before studying veterinary medicine, while one had started training as a surgical assistant and another had completed training as a veterinary assistant.

Of a total of 359 questionnaires submitted from veterinarians, 197 were excluded due to incompleteness. For the final evaluation, 162 questionnaires by veterinarians were analysed, with a total of 170 students assessed within elective practical training. Of the 162 veterinarians, 160 provided information on their age, the median was 48 years (minimum 25 years, maximum 75 years). 70% (*n* = 113) stated that they were female and 30% (*n* = 48) were male. One person did not specify gender.

The interviews with a total of six veterinarians took place between October 2024 and December 2024 and lasted between 7:22 min and 21:35 min. Five of the six participants were female and one was male. The ages of the veterinarians ranged from 30 years to 50 years. While one person had no specialization, four people stated that they had completed specialty training, while one person was in training for a specialty. The specializations mentioned included specialist veterinarian for bovine diseases, specialist veterinarian in anaesthesia, analgesia, intensive care and emergency medicine as well as the European Diplomate in Veterinary Anaesthesia (ECVAA), specialist veterinarian in small animals, specialist veterinarian in cattle and horses and further training for the European Diplomate in Veterinary Neurology (ECVN). Three of the institutions specialize in the treatment of small animals, one in small animals and small mammals, another in farm animals and another in the treatment of large and small animals, with large animals including both farm animals and horses. Four of the participating veterinarians work in clinics, three of which are university-based, while the remaining two work in practices.

### 3.2 Practical training specific information and supervision

Of the 124 practical training periods assessed by students, 30% (*n* = 37) were intramural practical training periods. The majority (60%, *n* = 75) were extramural practical training periods. For twelve practical training periods (10%), no indication was given as to whether it was an intramural or extramural practical training. Most students completed elective practical training in a clinic for small animals (42%), horses (18%) and livestock (16%). The next most popular option was practical training in mixed practices (14%). Only a few students mentioned a clinic for small mammals (2%) or other animals (6%). Most of the participating veterinarians came from small animal clinics (50%). Next came mixed practices (15%) and other facilities (13%). The smallest proportion was made up of horses (6%), livestock (6%) and small mammals (5%).

**Figure 1:**
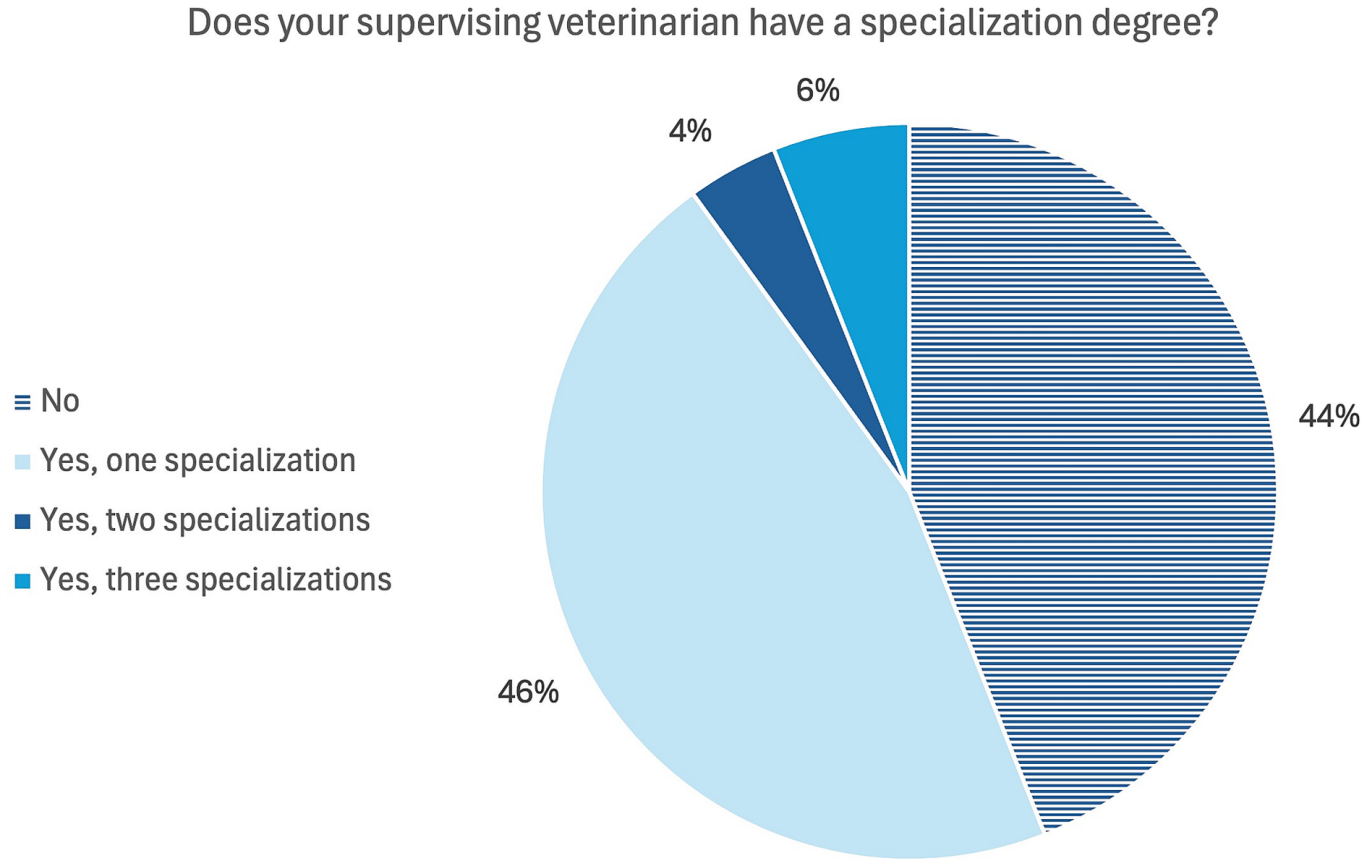
Online survey of students in practical year in Germany. Question: “Does your primary supervising veterinarian have a specialization degree?” (*n* = 124).

**Figure 2:**
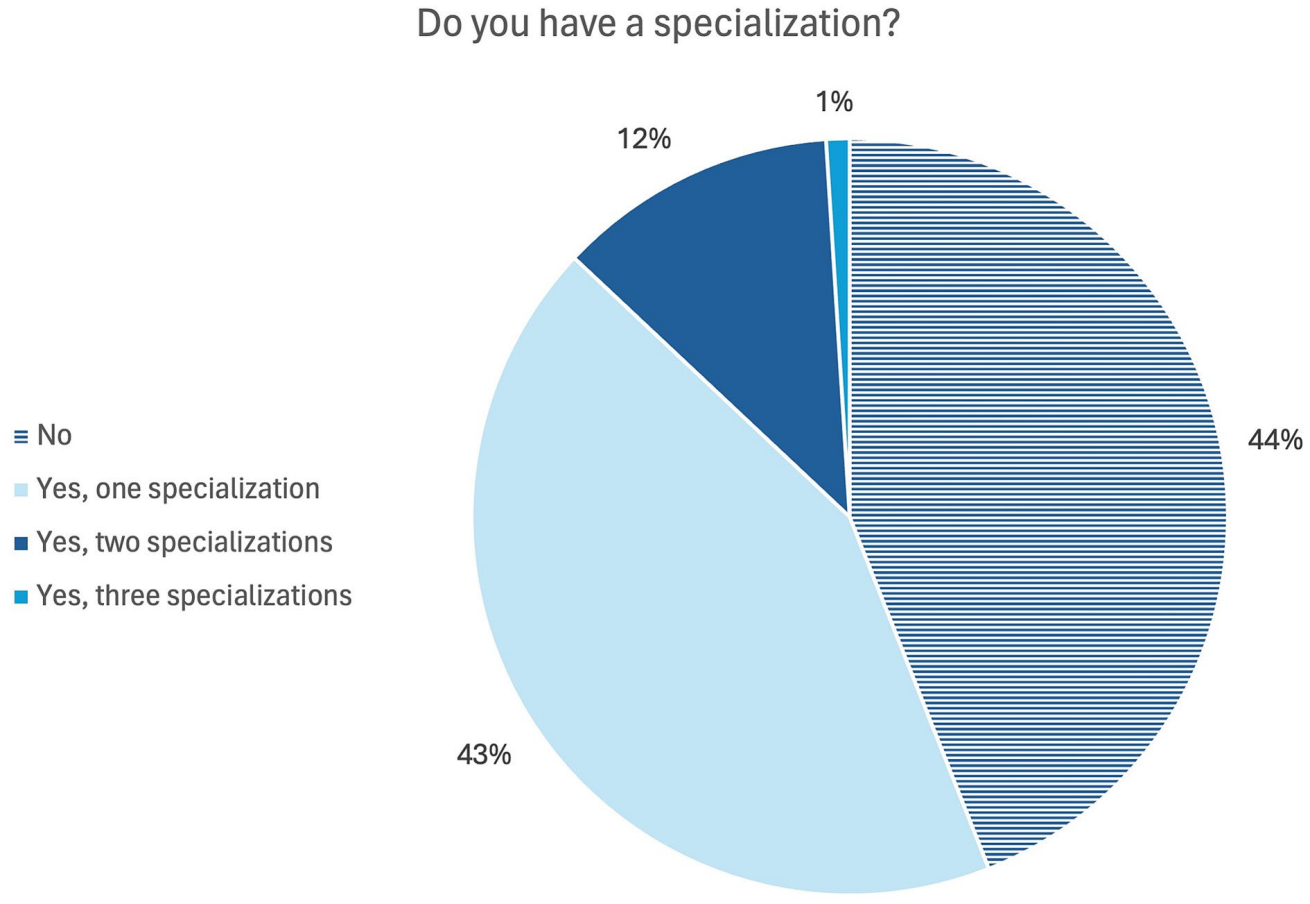
Online survey of veterinarians in Germany. Question: “Do you have a specialization?” (*n* = 162).

In more than half of the elective practical training, the primary supervising veterinarian had at least one specialization (Figure 1) according to students responses. The most frequently mentioned specialization (*n* = 42) was the national veterinary specialist. In terms of veterinary specialties, horses and small animals were mentioned most frequently and surgery and internal medicine were the most common specialties. Also, the veterinarians were asked whether they had any specialist training, with multiple answers being possible. The results are shown in Figure 2, compared to the students’ answers (Figure 1). More than half of the veterinarians (*n* = 90) have at least one specialization. The specialization in an area was mentioned most frequently. Species-specific, small animals and small animals and small mammals and the specialty relating to surgery and neurology were mentioned most frequently.

In the subsequent section of the survey, the objective was to ascertain the entity responsible for the supervision of students during their practical training. The survey allowed multiple options when multiple qualified individuals were involved in the supervision. This is shown in Table 1 in direct comparison with the information provided by the veterinarians.

**Table 1:** Question “Who is involved in the supervision of students?” left: Online survey of practicing veterinarians in Germany (*n* = 162) right: Online survey of students in practical year in Germany (*n* = 124).

Veterinary professionals	Answers from veterinarians	Answers from students
Veterinarian, Assistant veterinarian	94% (*n* = 153)	94% (*n* = 116)
Veterinary specialist	60% (*n* = 98)	66% (*n* = 82)
Veterinarian with additional qualification	29% (*n* = 47)	35% (*n* = 44)
European Diplomate	22% (*n* = 36)	27% (*n* = 37)
Veterinary paraprofessional	59% (*n* = 95)	65% (*n* = 81)
Other (e.g., Physiotherapist, Laboratory assistant)	-	29% (*n* = 37)

Details on the supervision structures were provided in more detail during the interviews with veterinarians. The interviewees stated that in principle, all employees who have contact with the students are responsible for supervising them during their elective practical training. This includes veterinarians as well as veterinary assistants, research assistants and doctoral students. However, there are often main contact persons in the institutions who are responsible for organization and tasks such as briefing and meetings. Overall, the supervision of students is a joint task in which higher-level organization and daily supervision by local staff work together. Supervision is influenced by a combination of professional responsibilities, personal interest and organizational framework conditions. Many of the people with primary responsibility are part of the practice or clinic management or take on management tasks, which means that supervision is part of their field of activity. Other participants are involved in professional politics and enjoy participating in the teaching and training of students. Overall, supervision is perceived as an integral part of their professional activity.

**Figure 3:**
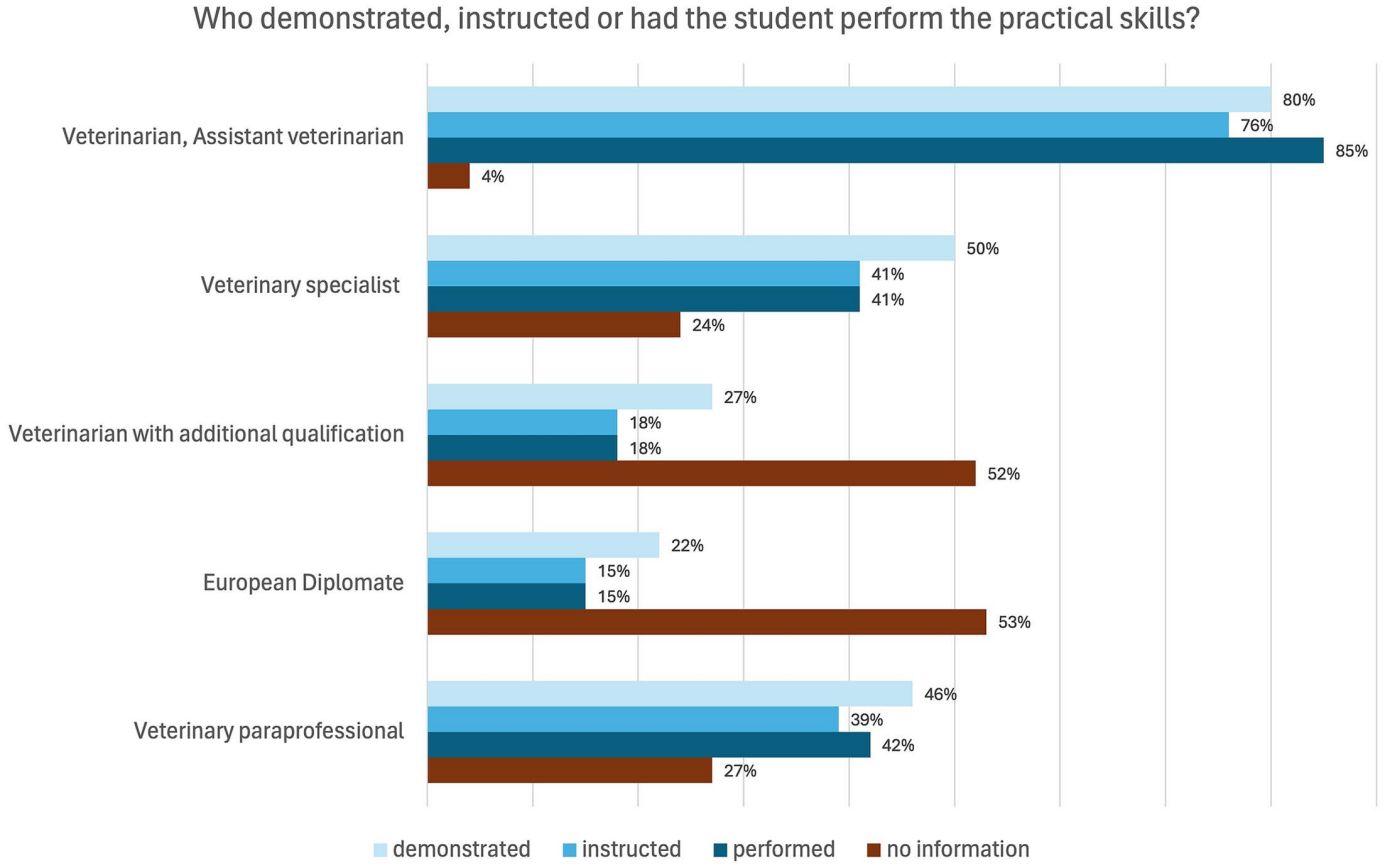
Online survey of students in practical year in Germany. Question: “Who demonstrated, instructed or had the student perform the practical skills?” Multiple Answer Format (*n* = 124).

For the majority of elective practical training periods (81%, *n* = 100), students stated that many practical activities could be carried out. In 41% (*n* = 51) of the practical training the answer was “Strongly agree,” and in 40% (*n* = 49), the answer was “Somewhat agree.” 22 students (18%) answered the question with “Somewhat disagree” and two students (2%) answered “Strongly disagree.” The interviews also revealed that practical activities could be carried out in most elective practical training periods, but the scope and type of activities varied greatly. Existing knowledge or previous training, the time of the practical training, the type of animal and the commitment of the students were identified as possible influencing factors. Routine tasks in particular, such as taking blood samples or administering medication, were entrusted to be carried out by the students. When asked if they could carry out many practical activities, the students replied: “*Yes, definitely, in all of my practical training*,” “*Very different. But it was based a bit on what I had already done before*.,” “*So it was more auxiliary work*.,” “*More in the livestock sector than in the small animal and equine sector*.” In the survey, students were also asked who was generally involved in teaching practical skills. Four students (3%) did not provide any information. In 114 placements (92%), the veterinarian and the assistant veterinarian were named. In 74 placements (60%), a veterinary specialist was involved in teaching the practical skills. A specialist veterinarian with an additional qualification was named in 32 placements (26%) and a European Diplomate in 28 placements (23%). Veterinary paraprofessionals were involved in 53 elective practical training (43%) and students stated “other” in 12 practical training periods (10%). Figure 3 presents the results for the question “Who demonstrated, instructed and had the student perform the practical skills?.” In order to clarify who shows, instructs or entrusts performance of tasks, multiple answers were possible for all supervisors in this question. In this figure, the ´no information´ category was selected when a particular professional group was either absent from the placement or not involved in instructing practical skills. Therefore, this category does not necessarily indicate an absence, but rather a lack of involvement in practical training from the students´ perspective.

**Figure 4:**
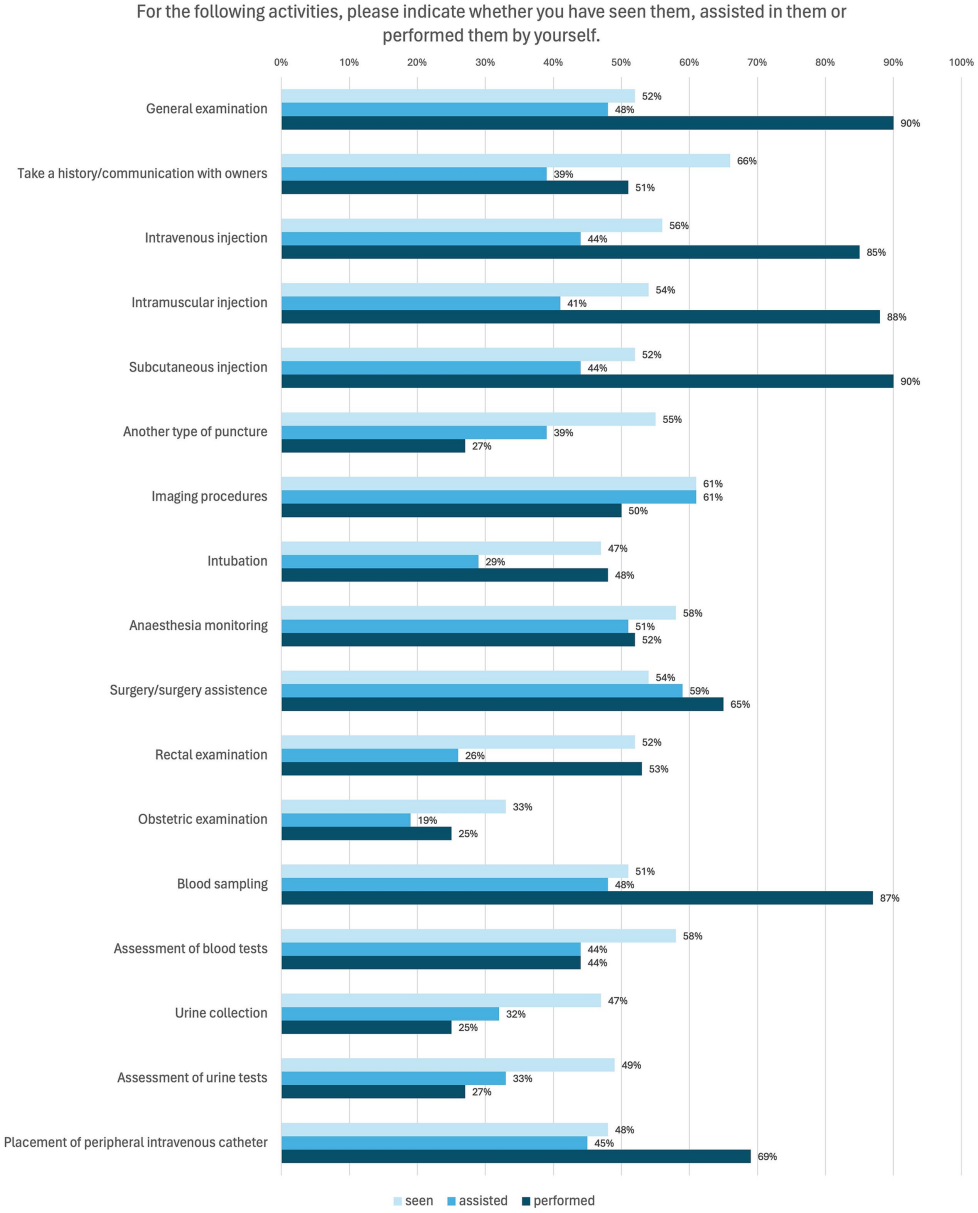
Online survey of students in practical year in Germany. Question: “For the following activities, please indicate whether you have seen them, assisted in them or performed them by yourself.” Multiple Answer Format (*n* = 124).

The students had the opportunity to indicate whether they had seen, assisted with or carried out a selection of practical skills. Multiple answers were possible if all of the above applied. The results are visualized in Figure 4. It shows that injection techniques, blood sampling and general examinations were performed most frequently, whereas communication with clients or specialized diagnostics were performed less frequently.

Three students (2%) negated the question “Were you able to take the opportunity to discuss clinical cases during the elective practical training (signalement, predispositions, clinic, laboratory changes, diagnostics, differential diagnoses, therapy, prognosis).” Clinical cases were discussed with the veterinarian in 105 placements (85%). In 79 placements (64%), assistant veterinarians played a role in the discussion of clinical cases, in 66 placements (53%) the veterinary specialist and in 30 placements (24%) the veterinarians with additional qualifications. In 26 placements (21%), it was stated that clinical cases were discussed with a European Diplomate.

In the next step, the students were asked about the quality of the above-mentioned meetings in which clinical cases were discussed. In 45% (*n* = 56) of the elective practical training, the quality was rated as “very good” and in 41% (n = 51) as “good.” The question was answered with “unsatisfactory” in 10% (*n* = 12), while no answer was given five times (4%). Subjective experiences from the interviews revealed that specialists tended to convey theoretical knowledge better than assistant veterinarians due to their higher level of expertise. However, the assertion was also made that many years of professional experience can also lead to a high level of knowledge and greater professional competence even when no specialization was achieved.

In general, 46% (*n* = 57) of the elective practical training were rated as “very good,” 39% (n = 48) as “good” and 15% (*n* = 18) were rated as “unsatisfactory.” This question was not answered in the evaluation of one practical training period (1%).

In a multiple answer format, the students named “teaching and good supervision” (40%, *n* = 49), “range of treatments” (35%, *n* = 44) and, at 30% (*n* = 37) each, “reputation” and “possibility for future employment” as the primary factors influencing their choice of elective practical training. Other reasons were “Working atmosphere” (27%, *n* = 34), “Other” (23%, *n* = 28), “Previous experience in the company” (22%, *n* = 27), “Attractiveness of the environment” (21%, *n* = 26), “Personal factors” (18%, *n* = 22) and “Compensation for expenses” (14%, n = 17).

In most elective practical training (69%, *n* = 86), students stated that no preliminary discussion had taken place in which learning objectives, expectations and framework conditions were discussed and defined. In 27% (*n* = 34), such a preliminary discussion took place. No information on this question was provided in four (3%) of the elective practical training assessed. The interviews also confirm that the majority of elective practical training did not have a formal preliminary interview. In most cases, introductory events were held by the veterinary educational establishments, or the framework conditions were defined by the practical training provider. The students’ experiences also show that a definition of the objectives in advance would have been helpful in many cases. However, students show understanding for the fact that the supervision of the students has to be completed alongside their everyday work. In addition to information on a possible preliminary discussion, the interviews also provided insights into the review of learning progress during the elective practical training. While the veterinary educational institutions use checklists and logbooks, extramural practical training tend to use verbal agreements, team meetings and staff discussions to evaluate the students’ learning progress.

In more than half of the elective practical training periods (52%, n = 65), the students rated the importance of the supervising veterinarian’s specializations as “important” (40%, *n* = 49) or “very important” (13%, *n* = 16) when selecting the placement. In 27% (*n* = 33) of the placements, further training was rated as “not very important” and in 17% (*n* = 21) as “not important.” In five (4%), no information was provided on this question. The interviews also revealed a variable prioritization of specializations when choosing a work placement. Students whose interests focus on special subject areas and those who are also aiming for postgraduate training formulated the importance of specializations of the supervising veterinarians.

The question of whether the students felt differently supervised by veterinarians with different qualifications was answered by 14% (*n* = 17) with “Strongly agree,” and by 26% (*n* = 32) with “Somewhat agree.” In 18% (*n* = 22) of the practical training, the students did rather not feel differently supervised (“Somewhat disagree”), and in 27% (*n* = 33) not at all (“Strongly disagree”). In 20 (16%), no information was provided on this question.

In a free text response, the students had the opportunity to explain how they felt differently supported: “*Depending on level of knowledge and professional experience*,” “*Assistant veterinarians have taken more time*.” “*Specialist: more detailed and specific discussion of cases*.” Above all, the specialists were attributed a high level of professional competence and a more competent demeanor. However, as they often held senior positions and were therefore not necessarily responsible for supervising the students, they tended to have less time. In contrast, assistant veterinarians had more time or took more time and were more approachable, friendly and understanding. However, due to their limited experience, they were often still uncertain, which is why only superficial explanations were provided for questions. Overall, the quality of the training depends on the level of knowledge and professional experience. Individual motivation and willingness to teach and supervise students were also identified as influential factors. These statements are supported by the interviews, in which the quality of training is made dependent on professional experience and level of knowledge, whereby a high level of knowledge often goes hand in hand with specialization. It is described that good supervision was provided by both specialized and non-specialized veterinarians if they were committed and accessible. Overall, respondents seem to believe that a balanced selection of placements, including both specialized veterinarians and general practitioners, is most beneficial for training.

When asked about the focus of the students’ training, the veterinarians were able to choose between theoretical knowledge, practical skills and professional behavior, with multiple answers being possible. 96% (*n* = 156) of the veterinarians stated that the focus of the students’ training during the practical year was on teaching practical skills. 71% (*n* = 115) focused on the teaching of professional behavior and 47% (*n* = 76) on the teaching of theoretical knowledge. Observational statistics can be used to determine the predictive value of the probability that the answer “theoretical knowledge” will be given. The highest probability is for the specialist veterinarian (53%), followed by the European Diplomate (52%). The probability that a veterinarian without specialization answer the question with “theoretical knowledge” is 42%. No predictive values can be determined for answering the question with “practical skills,” as the majority of veterinarians (96%) gave this answer. The probability of a European Diplomate to answer the question with “professional behavior” is 88%, for a veterinarian without specialization 72%.

The interviews with the veterinarians make it clear that, regardless of specialization, value is placed on basic training and the acquisition of Day One Competences: “*I’m more a fan of: first you have to be able to perform the basic procedures on all animal species*,” “*It’s important to me that people receive comprehensive training*.” Broad-based knowledge is seen as an indispensable foundation for later professional practice.

53% (*n* = 86) of veterinarians answered “Yes” to the question “Is there a preliminary discussion in which learning objectives, expectations and framework conditions of the elective practical training are discussed and defined?,” while 41% (*n* = 66) stated that there was no preliminary discussion. 6% (*n* = 10) did not answer this question.

### 3.3 Assessment of skills

**Figure 5:**
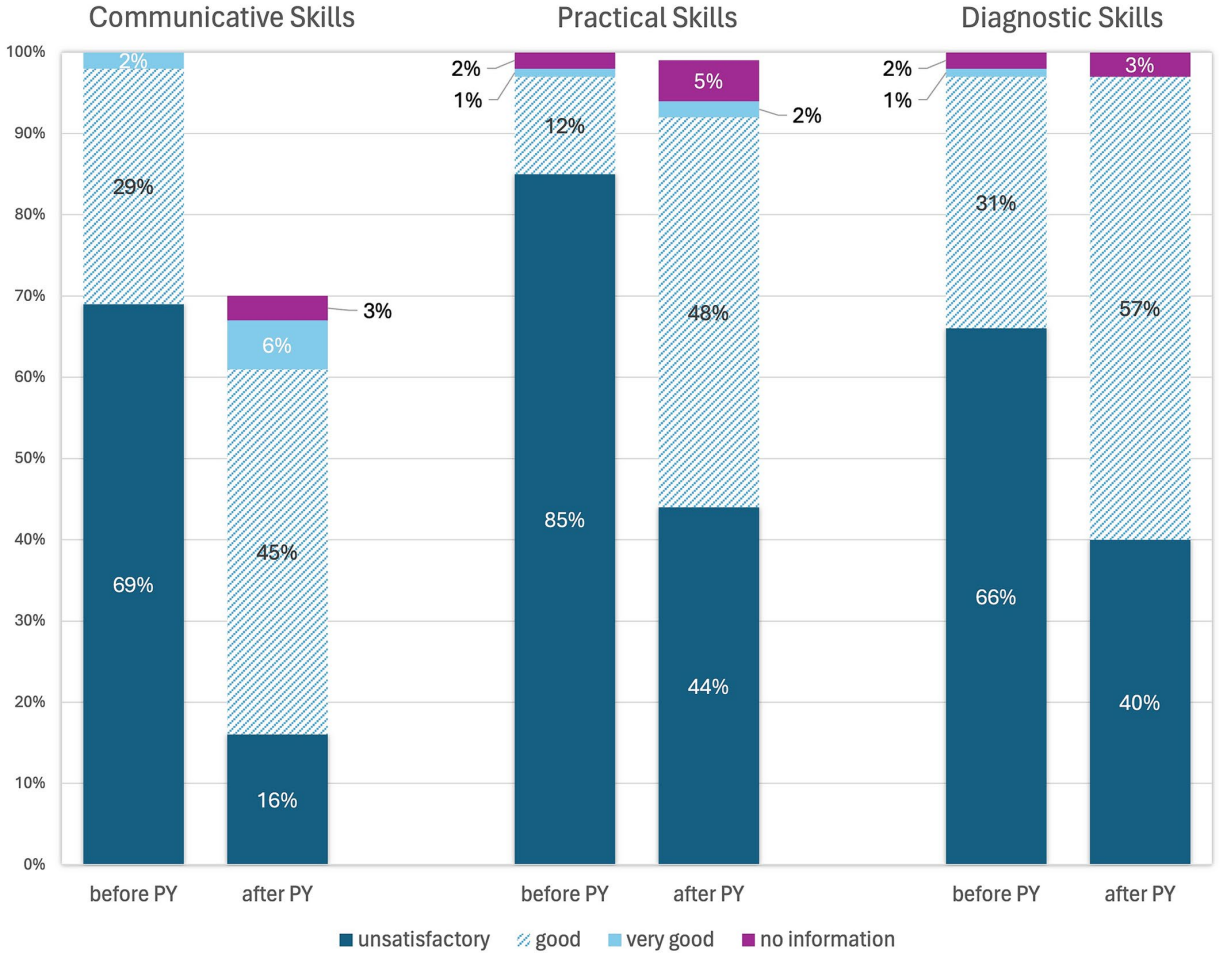
Online survey of students in practical year in Germany. Questions:—before Practical Year (PY): “How well do you feel prepared by your study period (before PY) for your career entry?”—after Practical Year (PY): “How would you rate your skills at present (after PY)?” (*n* = 99).

To assess their own skills, students were asked questions about their communication, practical and diagnostic skills. The questions “How well do you feel prepared by your study period (before the practical year) for your career entry in terms of your communicative/practical/diagnostic skills?” and “How would you rate your own communicative/practical/diagnostic skills at the present time (after completing the practical year)?” are shown together in Figure 5 for the purpose of better comparability.

Most students (76%, *n* = 75) stated that the practical year helped them to improve their communication skills (“Strongly agree” 21%, *n* = 21; “Somewhat agree” 55%, *n* = 54). 18% (*n* = 18) answered this question with “Somewhat disagree” and 5% (*n* = 5) with “Strongly disagree.” One person gave no answer. Regarding practical skills, 87% (*n* = 86) of students stated that the practical year had helped them to improve their practical skills (“Strongly agree” 35%, *n* = 35; “Somewhat agree” 54%, *n* = 53), while 10% (*n* = 10) answered “Somewhat disagree” and 3% (*n* = 3) “Strongly disagree.” In terms of improving diagnostic skills, the practical year helped 87% (*n* = 86) students (“Somewhat agree” 61%, *n* = 60; “Strongly agree” 26%, *n* = 26). 10% (*n* = 10) stated that the practical year did not help (“Somewhat disagree”) and 3% (*n* = 3) that it did not help at all (“Strongly disagree”).

**Figure 6:**
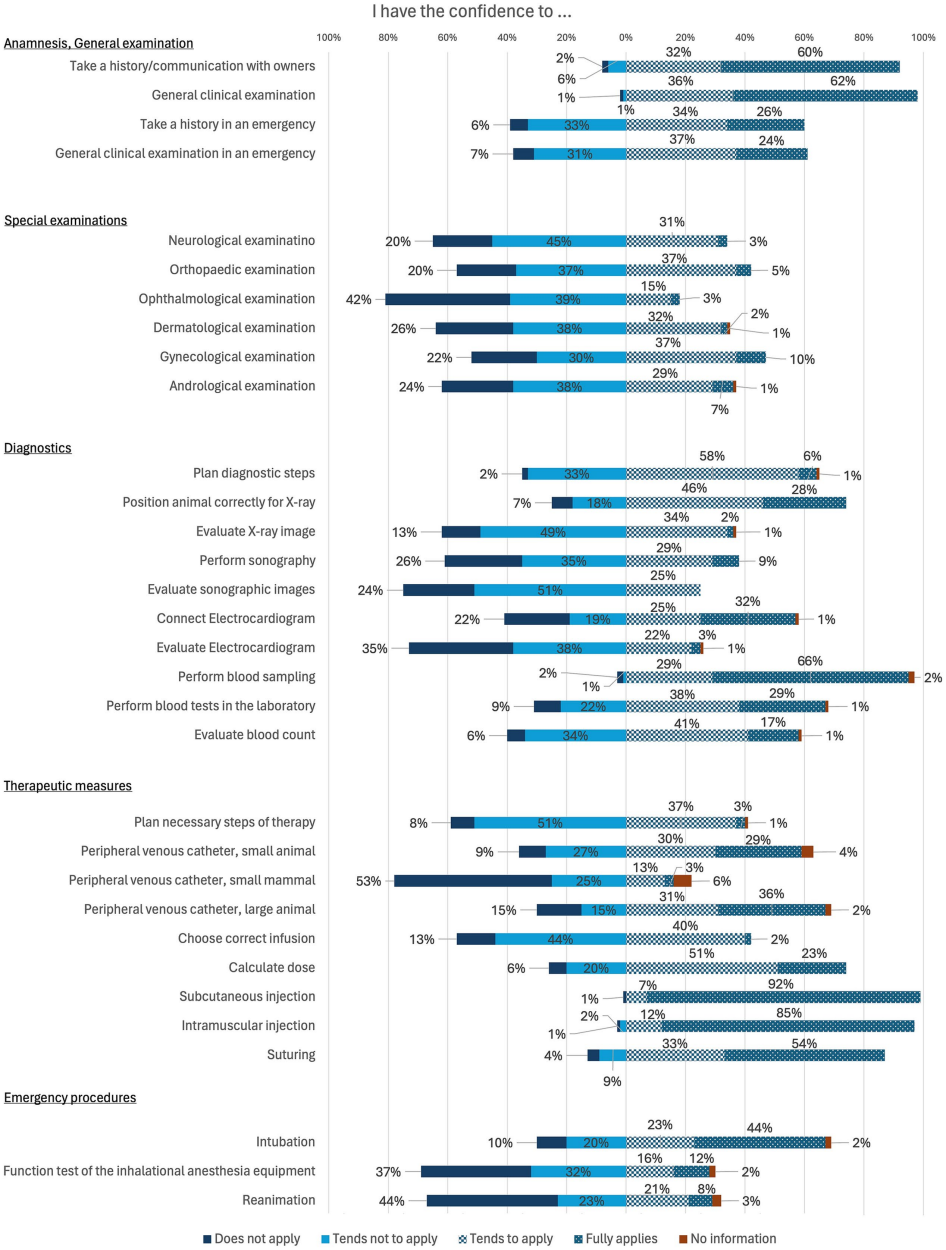
Online survey of students in practical year in Germany. Question: “I have the confidence to …” (*n* = 99).

In the final part of this section, students were asked to provide a self-assessment regarding the performance of specific activities. The corresponding results are shown in Figure 6. Students are particularly confident in basics such as taking a history, general clinical examination and basic diagnostics and therapeutic measures such as performing blood sampling and injections. They do not yet feel as confident in special examinations and some diagnostics such as evaluate X-ray images, perform sonography and evaluate sonographic images or evaluate an electrocardiogram.

**Figure 7:**
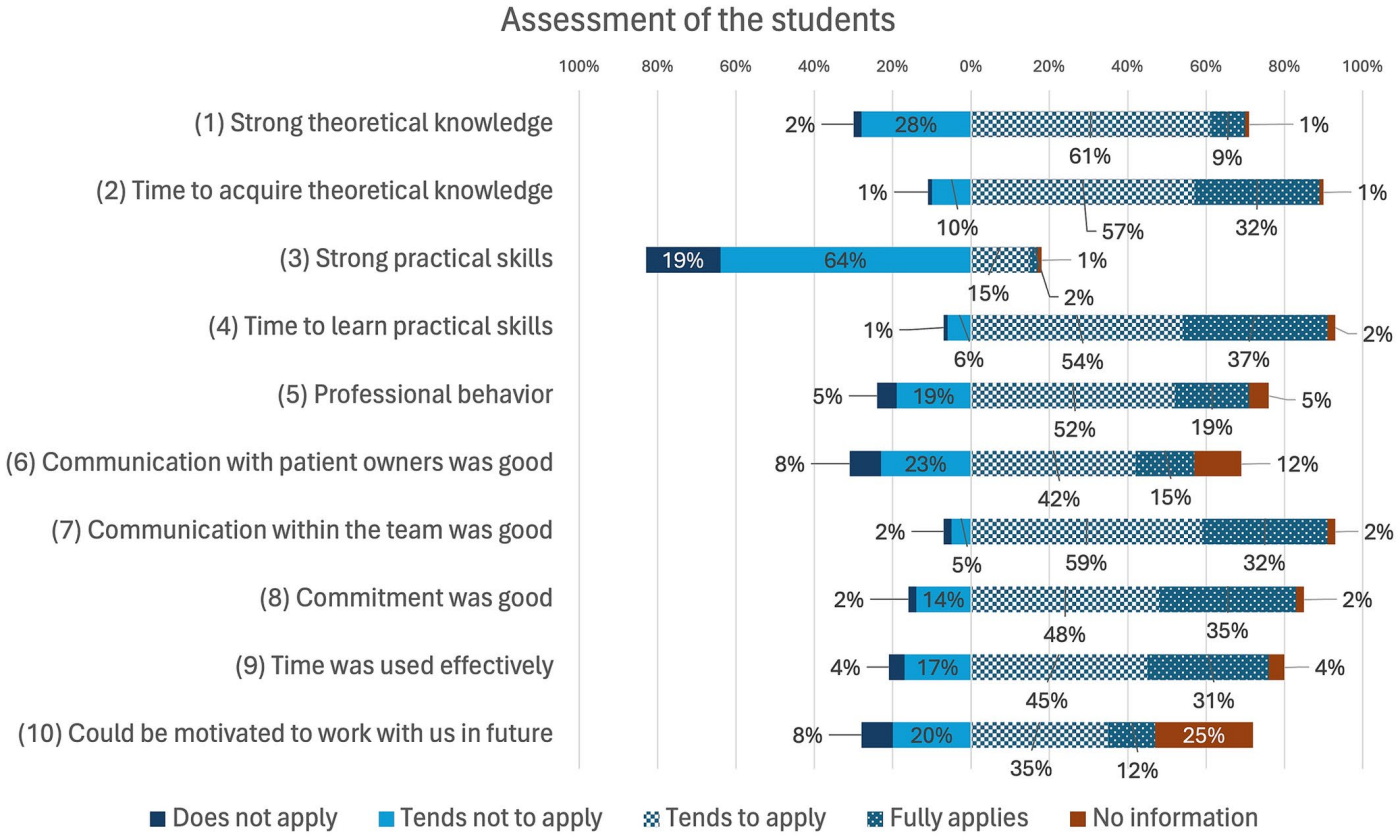
Online survey of veterinarians in Germany. Question: “Assessment of students” (*n* = 170).

The results of the section on student assessment of the veterinarians´ survey are shown in Figure 7. The students’ skills were predominantly assessed positively by the veterinarians. However, a deficit in practical skills was observed.

In the interviews, the assessment of the students shows a variability that can be attributed to individual differences as well as the stage of their studies or of the practical year (start or end of the year). When asked about the students´ skills, the veterinarians replied: “*I must say, there is a high degree of* var*iability.*,” “*Practical skills are highly dependent on how many practical trainings they have already completed*.,” “*I would rate theoretical knowledge as average, because university knowledge is much broader than the knowledge we need in the practice*.,” “*Communication is more of a personal thing*.” In terms of theoretical knowledge, students are generally well trained, although their knowledge is often broad and not always relevant to practice. Immediately after graduation, practical skills are often still poorly developed, although this improves as the practical year progresses. Communication is highly dependent on the type of student and varies depending on their personality. Overall, communication skills are rated as good. Communication skills are also rated as particularly important and prioritized over theory and practical skills. As practical skills are mainly learned in the practical year and in the first years of work, students should have well-founded theoretical knowledge after graduation. According to the veterinarians, the assessment and evaluation of students’ learning success is primarily qualitative in the form of feedback discussions and observations. There are usually no formal guidelines for assessing learning progress. Some institutions use the logbooks of veterinary education establishments or guidelines from institutions such as the bpt (Federal Association of Practicing Veterinarians).

### 3.4 Student satisfaction

**Figure 8:**
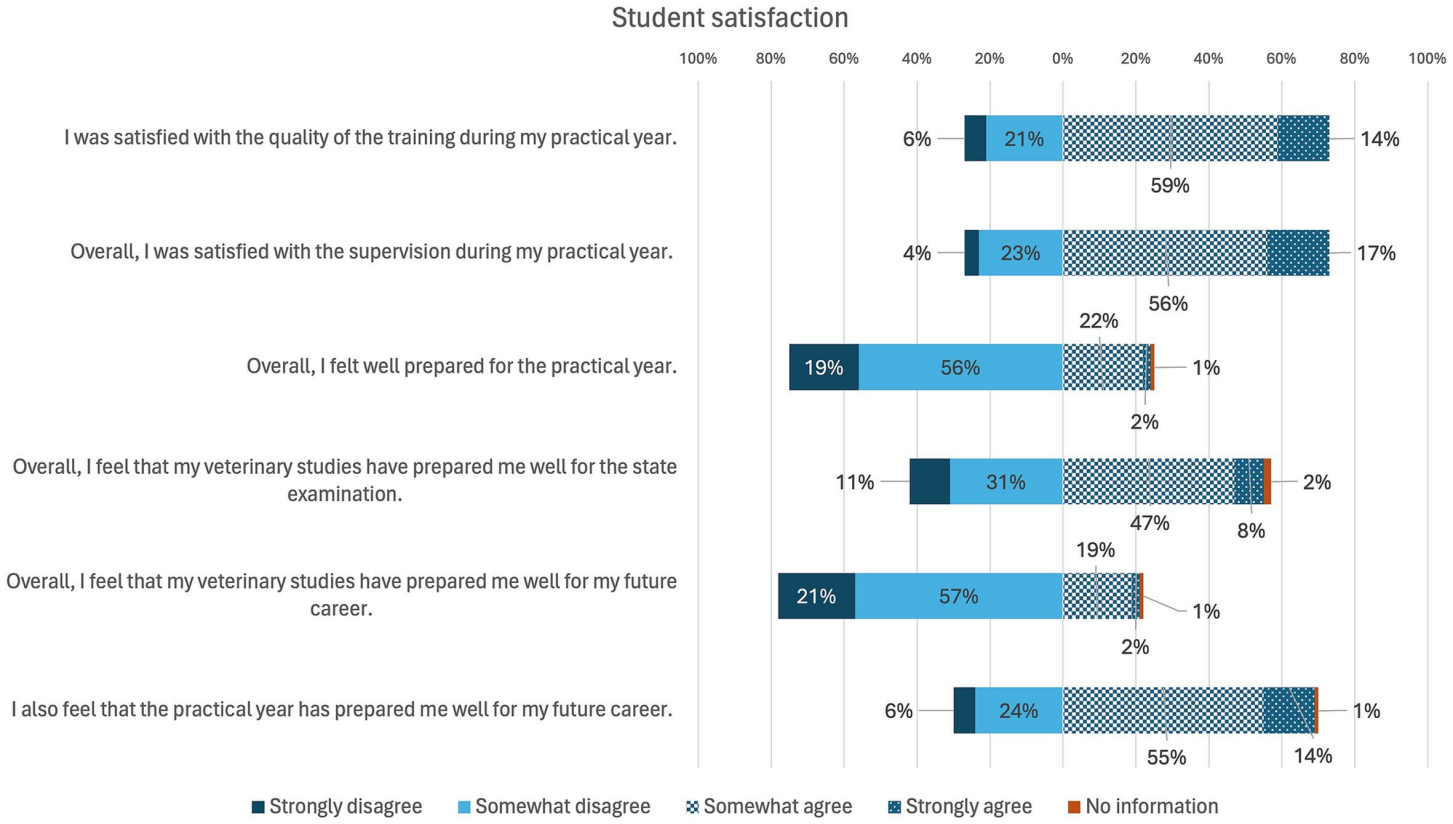
Online survey of students in practical year in Germany. Question: “Student satisfaction” (*n* = 99).

The results of student satisfaction are visualized in Figure 8. The majority of students (*n* = 72) were satisfied with the quality of training during the practical year, as well as with the overall supervision during the practical year. At 74, the majority of students did not feel well prepared for the practical year during their studies. The majority of students (*n* = 77) had the feeling that they were not well prepared during their studies for their future work. However, 68 students felt that the practical year prepared them well for their future career.

## 4 Discussion

In the current study the quality of clinical training in the practical year and the influence of specialization of teaching veterinarians was evaluated. Overall, valuable findings were obtained to identify trends for improving teaching and clinical training, which can inform future guidelines for students on how to choose and organize their elective practical training. In addition, the relevance of the practical year in the clinical training of students became evident.

Despite the relatively small sample size, heterogeneous distributions were shown and trends identified. According to statistics in Germany, 87% of female students were enrolled in veterinary medicine courses in the winter term of 2023/2024 (35). With 92% female students taking part in this survey, the proportion of participating female students in this study are only slightly higher and reflect therefore the overall gender distribution in studies of veterinary medicine. In the survey of veterinarians, the gender distribution of this study (70% female veterinarians) is also similar to the overall distribution according to statistics (71% female veterinarians) (35).

Most participating veterinarians focus on teaching practical skills in clinical training to avoid deficits in practical skills as identified in the assessment of students’ skills before the practical year is taught (Figure 7). The individual interviews show that the deficit in practical skills is not viewed negatively, as the practical year is intended for learning these skills. Communication and theoretical knowledge are more important to veterinarians than practical skills, especially at the beginning of the practical year. Good practical skills are mainly observed in students with prior professional education such as training as a veterinary assistant. This was also observed in earlier studies (36). Working as a student assistant during their studies also means that these students are better prepared in terms of their practical skills. The time in the practical year (start, end of the year), when the practical training period takes place, also plays a role in how students are performing. The veterinarians’ assessment of the students’ practical skills is in line with the students’ self-assessment. Before the practical year, most students felt that they were not satisfactorily prepared for their career entry in terms of their practical skills (Figure 5). It should be noted that students tend to rate the learning of practical skills as more important than veterinarians (37). While only a small proportion of students rated their practical skills as good or very good before the practical year, this proportion increased after completing the practical year. Furthermore, the majority of students stated that the practical year helped them to improve their practical skills. In other studies, it was demanded that practical-clinical training should be increased (5, 6, 9–11), a fact confirmed by the veterinarians in the current study. The improvement in practical skills in the students’ self-assessment is a further indication. However, the individuality of the practical year can lead to resentment and supports the demand for greater standardization of elective clinical training periods. Overall, not all students trust themselves to carry out special examinations, diagnostic measures or emergency measures even after the practical year (Figure 6). However, it should be noted that this is a subjective assessment of skills that does not correspond to the actual competence (17). Students in human medicine also tend to have higher expectations regarding their clinical skills (38). In addition to practical skills, communication is also defined as a Day One Competence (3). Students’ communication skills were prioritized highest by veterinarians in this study and both communication with patient owners and communication within the team were predominantly rated positively (Figure 7). Overall, most students felt that the practical year had helped them to improve their communication. The high expectation of veterinarians for communication skills confirms the relevance of communication in the veterinary profession (39–41). Nevertheless, a stronger implementation of communication teaching, e.g., in the form of communication training with actors during the course of study, is necessary (41) in order to prepare students for situations in everyday clinical practice even before the practical year. And although the use of logbooks can promote the acquisition of skills (5), previous studies show that guidelines are rarely used or logbooks are incompletely filled out (12, 13, 17). In this study, too, more emphasis is placed on individual support for skills acquisition in the form of feedback discussions, particularly in extramural practical training. Though some institutions address common issues and concerns associated with extramural practical training periods (42), other veterinary education establishments are still challenged how to best prepare their students (43).

The fact that students observe an improvement in their Day One Competences during the practical year (44), but practicing veterinarians predominantly rate these skills as average for those entering the profession (36), raises the question of whether the expectations of veterinary educational establishments, students and practicing veterinarians regarding existing skills match (17). Inconsistencies between the expectations of both sides can result in an impairment of the students´ clinical training (37). According to this study, the variability and individuality of the practical year also play a role in clinical-practical training. The time period, animal species, students’ own initiative and veterinarians’ motivation influence the experience gained. Thus, according to students, there were more opportunities to carry out practical activities in the livestock sector and with general practitioners. A scoping review on rural placements of health students identified barriers and enablers to learning highlighted importance of interpersonal factors, learner engagement and the supervisor’s role (45). Similarly, within our study enablers such as a proactive attitude, the commitment of the students and their own initiative were emphasized as beneficial aspects to improve the experience and outcomes during the practical year. This is observed by both groups, the veterinarians and the students themselves. To adjust expectations, a preliminary discussion should take place in which learning objectives, expectations and framework conditions are defined and agreed upon by both sides as already suggested by the bpt (46, 47). While most students stated that no preliminary meeting took place, the majority of veterinarians stated that preliminary discussions were held. However, some students reported in the interviews that a preliminary interview had taken place, in which only the framework conditions were defined by the elective practical training provider. Students increasingly expressed the desire for such a preliminary discussion. In this way, students know what is expected of them, to what extent they should and may contribute, they can be integrated into everyday clinical practice in a more targeted manner and a more successful elective practical training experience can be guaranteed for students and practical training providers.

According to both, students and veterinarians, more than half of the veterinarians have at least one specialization (Figures 1, 2). In the interviews, some students suggested that specialization was particularly necessary or important in the small animal and equine sectors, but less important in the livestock sector. In livestock practical training, students placed more value on professional experience and area of practice than on existing specializations. In addition to veterinarians and assistant veterinarians and veterinary specialists, veterinary paraprofessionals were also responsible for teaching practical skills. In the interviews it became evident that veterinary assistants are able to teach Day One Competences, especially during inpatient care. The role of veterinary paraprofessionals in teaching practical skills is also described in earlier studies (26). This is also evident in the general assessment of supervision (Table 1). The slight discrepancies between the reports of students and veterinarians may be explained by the fact that the veterinarians refer to officially assigned supervisory roles, while students report the supervision they actually experienced, which may also include informal or unassigned support by staff members. The relevance of the veterinarians’ specializations for the students is emphasized for choosing a work placement. More than half of the students considered veterinary specialization to be important or very important. Multiple aspects affect the search for and decision where to engage in the elective practical training period. Human medicine students reported that proximity, financial incentives and the subject where most important when choosing their placement (48). Even though only fewer than half of the students participating in our study felt that they received different care from veterinarians with different qualifications, specialization is associated with the expectation that students may receive better clinical training. The differences in supervision recorded in this study mainly related to specialists and assistant veterinarians and could not be determined in general between specialists and non-specialists. The assistant veterinarians were credited with having more time and taking more time for teaching. Students within this study mentioned that clinical cases could be discussed in more detail with specialists and theoretical knowledge could be acquired, whereas the assistant veterinarians were often only able to provide superficial explanations to questions due to their limited experience. The specializations of veterinarians have an influence on the quality of clinical training in that they are associated with a high level of knowledge and can therefore guarantee a high level of specialist training. However, the lack of specialization does not preclude a high level of knowledge. Veterinarians with many years of professional experience can also have a high level of knowledge. Further training that does not involve obtaining a title or additional designation can also increase the level of knowledge. In the interviews with veterinarians, the specialists also explained that they focus on training of basic knowledge and skills. Specialization is more likely to have an impact if students show great interest for such a specialization in their future careers.

Regardless of specialization and professional experience, students and veterinarians noticed a very individual motivation for teaching and training of students. This also plays a major role in the quality of training, as students consider teaching and good supervision to be the most important factors when choosing a work placement for elective practical training. In his work, Scarletti also attributes the greatest influence on the benefits of practical training to supervision (49). However, the motivation of students is also described by veterinarians as highly variable. Ultimately, there is an interaction between the motivation of students and of veterinarians.

Overall, most of the practical training periods were rated as good or very good, which aligns with previous students’ evaluation of elective clinical practice periods (47). The qualitative assessment depended primarily on the quality of supervision and the motivation of the veterinarians as well as the opportunity to carry out practical activities. Although most students do not feel adequately prepared for their future careers, the practical year helped the majority of students to prepare themselves well for their future careers. The veterinarians’ expectations of the students mainly concern commitment, interest and curiosity. Students should receive basic training, which includes learning practical skills as well as recognizing clinical signs and diseases and knowing how to treat them. In order to improve learning, it has been shown in human medicine that feedback is an essential component (50). It is also clear from the interviews that students would like more constructive feedback that goes beyond the school grading system to improve their skills. Following this study, which aimed to provide a general overview of the perceived quality of elective practical training, future research could take a more detailed look at differences between institutions in order to identify specific areas for improvement and adapt curricula accordingly to enhance training quality. In addition, future studies could investigate the role of veterinary paraprofessionals as supervisors in elective practical training further. Their importance in student training was assumed beforehand, as reflected in the findings of this study. However, a more detailed exploration would provide valuable insights into their specific contributions.

One of the limitations of the study is the relatively small sample size, also diminished by a high proportion of incomplete surveys, which is why the statistical analysis must be viewed critically. Due to the voluntary participation in the survey, it can be assumed that the participants are particularly interested in the topic and that possible expectations can influence the answers. In addition, these are subjective assessments of skills that do not reflect the actual competence. To objectively assess the students’ skills, an objective structured clinical examination (OSCE) could be carried out with a larger sample based on this study. It should be noted that not only the self-assessment of practical skills is subjective, but also the responses regarding the presence of specialist qualifications among supervising veterinarians reflect the students´ personal perceptions. Uncertainty and inconsistency in answering the questionnaire regarding specialists’ qualifications were perceived, which revealed issues of understanding of postgraduate continuing education among veterinarians and students.

The results of this study suggest that the practical year effectively fulfils its intended purpose of providing students with hands-on training and enhancing their practical skills. Although the specialization of supervising veterinarians may influence students´ learning experiences and should therefore be considered when selecting a placement, the quality of clinical training does not depend solely on specialization. Experienced, motivated and committed veterinarians can also provide high-quality education and make a significant contribution to students´ professional development.

## Data Availability

The datasets presented in this article are not readily available because the data are not publicly available due to ethical and privacy restrictions, i.e., any data transfer to interested persons is not allowed without a specific formal contract. This contract is available to qualified researchers and will include guarantees of the obligation to maintain data confidentiality in accordance with the provisions of the German data protection law. Currently, there is no data access committee or another body who could be contacted for the data. However, for this purpose, a committee will be founded. This future committee will consist of the authors as well as members of the University of Veterinary Medicine Hannover and members of the funding institution. Interested cooperative partners who are able to sign a contract as described above may contact the corresponding author. Requests to access the datasets should be directed to C. Kleinsorgen, christin.kleinsorgen@tiho-hannover.de.
