# Supplementary material for: Clinical training in veterinary medicine: perspectives on quality and the role of specialists
Source: Front Vet Sci. 2025 Jun 25;12:1601783. doi: 10.3389/fvets.2025.1601783 (PMC12239136; doi:10.3389/fvets.2025.1601783)
Supplement: Supplementary file 1 [file Table_1.docx]

Supplementary Material

# Student survey

**Demographics**

• Year of birth

• Gender

- Male

- Female

- Other

- No information

• Did you have previous professional training?

- No, this is my first professional training.

- I have started another training/course.

- I have completed another training/course.

- No information

• Please name your started/completed training/course.

• At which location do you study veterinary medicine?

- University of Veterinary Medicine Hannover

- University of Berlin

- University of Gießen

- University of Leipzig

- University of Munich

- No information

**Practical year**

• Please indicate whether the internship was completed in a university clinic (intramural) or at a private clinic/practice (extramural).

- intramural

- extramural

- No information

• The internship was completed in a:

- Clinic for large animals (Livestock)

- Clinic for large animals (horses)

- Clinic for small animals

- Mixed large and small animals

- Small mammals

- Other

- No information

• How many veterinarians work in the practice/clinic?

• Does your supervising veterinarian have a specialization degree?

- No (Veterinarian, Assistant veterinarian

- Yes, Veterinary specialist

- Yes, Veterinarian with additional qualification

- Yes, European Diplomate

- Other

• In which specialty is your supervising veterinarian specialized?

• Please rate the percentage of time you were supervised by each person.

- Veterinarian, Assistant veterinarian

- Veterinary specialist

- Veterinarian with additional qualification

- European Diplomate

- Veterinary paraprofessional

- Physiotherapist

- Laboratory assistant

- Office assistant

• Were you involved in the emergency service?

- No

- Yes, once a week

- Yes, more than once a week

- Yes, less than once a week

- No information

• Were you able to carry out many practical activities?

- Yes, definitely

- Yes, rather

- No, rather not

- No, not at all

- No information

• Who was responsible for teaching practical skills?

- Veterinarian, Assistant veterinarian

- Veterinary specialist

- Veterinarian with additional qualification

- European Diplomate

- Veterinary paraprofessional

- Other

• Who demonstrated, instructed or had the student perform the practical skills?

- Veterinarian, Assistant veterinarian

- Veterinary specialist

- Veterinarian with additional qualification

- European Diplomate

- Veterinary paraprofessional

• For the following activities, please indicate whether you have seen them, assisted in them or performed them by yourself.

- General examination

- Take a history/communication with owners

- Intravenous injection

- Intramuscular injection

- Subcutaneous injection

- Another type of puncture

- Imaging procedures

- Intubation

- Anaesthesia monitoring

- Surgery/surgery assistance

- Rectal examination

- Obstetric examination

- Blood sampling

- Assessment of blood tests

- Urine collection

- Assessment of urine tests

- Placement of peripheral intravenous catheter

• How would you rate the instructions for carrying out the practical activities?

- very good

- good

- unsatisfactory

- No information

• Where you able to take the opportunity to discuss clinical cases during the internship (signalement, predispositions, clinic, laboratory changes, diagnostics, differential diagnoses, therapy, prognosis)?

- Yes, with a veterinarian

- Yes, with an Assistant veterinarian

- Yes, with a Veterinary specialist

- Yes, with a veterinarian with additional qualification

- Yes, with a European Diplomate

• How do you rate the quality of the case discussions?

- very good

- good

- unsatisfactory

- No information

• How would you rate the internship in general?

- very good

- good

- unsatisfactory

- No information

• Why did you choose this internship?

- Teaching and good supervision

- Range of treatments

- Reputation

- Possibility for future employment

- Working atmosphere

- Previous experience in the company

- Attractiveness of the environment

- Personal factors

- Compensation for expenses

- Other

• Has a preliminary meeting taken place in which learning objectives, expectations and framework conditions were discussed and defined?

- No

- Yes

- No information

• How important is the specialization of the supervising veterinarian for you when choosing an internship?

- very important

- important

- not very important

- not important

- No information

• Did you feel different supervised by veterinarians with different qualifications?

- Yes, definitely

- Yes, rather

- No, rather not

- No, not at all

- No information

• Please describe to what extent you perceived the supervision provided by differently qualified staff to be different.

• Were the expectations of your internship fulfilled?

- Yes, definitely - Yes, rather - No, rather not - No, not at all - No information

- in terms of supervision and teaching

- in terms of the working atmosphere

- in terms of the range of treatments

- in terms of location and leisure activities

- in terms of the possibility to expand theoretical knowledge

- in terms of the possibility to acquire practical skills

- in terms of the possibility to improve communication skills

- in terms of other personal factors

**Self assessment**

• How well do you feel prepared by your study period (before practical year) for your career entry regarding your communication skills?

- very good

- good

- unsatisfactory

- No information

• Did your practical year help you to improve your communication skills?

- Yes, definitely

- Yes, rather

- No, rather not

- No, not at all

- No information

• How would you rate your communication skills at present (after practical year)?

- very good

- good

- unsatisfactory

- No information

• How well do you feel prepared by your study period (before practical year) for your career entry regarding your practical skills?

- very good

- good

- unsatisfactory

- No information

• Did your practical year help you to improve your practical skills?

- Yes, definitely

- Yes, rather

- No, rather not

- No, not at all

- No information

• How would you rate your practical skills at present (after practical year)?

- very good

- good

- unsatisfactory

- No information

• How well do you feel prepared by your study period (before practical year) for your career entry regarding your diagnostic skills?

- very good

- good

- unsatisfactory

- No information

• Did your practical year help you to improve your diagnostic skills?

- Yes, definitely

- Yes, rather

- No, rather not

- No, not at all

- No information

• How would you rate your diagnostic skills at present (after practical year)?

- very good

- good

- unsatisfactory

- No information

• I have the confidence to …

- Does not apply - Tends not to apply - Tends to apply - Fully applies - No information

🡪 Anamnesis, General examination

- Take a history/communication with owners

- General clinical examination

- Take a history/communication with owners in an emergency

- General clinical examination in an emergency

🡪 Special examinations

- Neurological examination

- Orthopaedic examination

- Ophthalmological examination

- Dermatological examination

- Gynecological examination

- Andrological examination

🡪 Diagnostics

- Plan diagnostic steps

- Position animal correctly for X-ray

- Evaluate X-ray image

- Perform sonography

- Evaluate sonographic images

- Connect Electrocardiogram

- Evaluate Electrocardiogram

- Perform blood sampling

- Perform blood tests in the laboratory

- Evaluate blood count

🡪 Therapeutic measures

- Plan necessary steps of therapy

- Peripheral venous catheter, small animal

- Peripheral venous catheter, small mammal

- Peripheral venous catheter, large animal

- Choose correct infusion

- Calculate dose

- Subcutaneous injection

- Intramuscular injection

- Suturing

🡪 Emergency procedures

- Intubation

- Function test of the inhalational anesthesia equipment

- Reanimation

**Student satisfaction**

- Yes, definitely - Yes, rather - No, rather not - No, not at all - No information

• I was satisfied with the quality of the training during my practical year.

• Overall, I was satisfied with the supervision during my practical year.

• Overall, I felt well prepared for the practical year.

• Overall, I feel that my veterinary studies have prepared me well for the state examination.

• Overall, I fell that my veterinary studies have prepared me well for my future career.

• I also feel that the practical year has prepared me well for my future career.

• If you would like to add anything else, you can do so here.

# Veterinarian survey

**Demographics**

• In which year where you born?

• What is your gender?

- Male

- Female

- Other

- No information

• In which year did you receive your license to practice medicine?

• How long have you been a practicing veterinarian?

• Do you have a specialization?

- No

- Yes, Veterinary specialist

- Yes, Veterinarian with additional qualification

- Yes, European Diplomate

- Other

• In which specialty are you specialised?

• In which institution do you work?

- Practice

- Clinic

- Other

- No information

• Which animals are treated in the practice/clinic where you work?

- Small animals

- Large animals (horses)

- Large animals (Livestock)

- Mixed large and small animals

- Small mammals

- Other

- No information

• How many veterinarians work in the practice/clinic where you work?

**Practical year**

• Who is responsible for supervising students in their practical year?

- Veterinarian, Assistant veterinarian

- Veterinary specialist

- Veterinarian with additional qualification

- European Diplomate

- Veterinary paraprofessional

• For me, the focus of training students in their practical year is on teaching them:

- theoretical knowledge

- practical skills

- professional behavior

• Is there an active search for interns (e.g. with the help of advertising)?

- Yes

- No

- No information

• Is there a preliminary meeting in which learning objectives, expectations and framework conditions are discussed and defined?

- Yes

- No

- No information

• What the practice/clinic offers interns?

- Working atmosphere

- Intensive supervision

- Wide range of treatments

- Possibility for future employment

- Compensation for expenses in form of accommodation and meals

- Other compensation for expenses

**Assessment of the students**

- Does not apply - Tends not to apply - Tends to apply - Fully applies - No information

• The student had a strong theoretical knowledge.

• The student had sufficient time and opportunities to acquire and deepen subject-specific theoretical knowledge during the internship.

• The student had strong practical skills.

• The student had sufficient time and opportunities to learn and improve subject-specific practical skills during the internship.

• The student emphasized professional behavior.

• The student´s communication with patient owners was good/professional.

• The student´s communication within the team was good.

• The student´s commitment and dedication to the internship was good.

• The student has used the time available effectively.

• The student could be motivated for future cooperation.

• If you would like to add anything else, you can do so here.

**3 Interview guidelines students**

Topic: Evaluation of the supervision and clinical training of students during the practical year as well as the influence of specializations of the supervising veterinarians

Aim: Recording of subjective experiences with regard to the supervision of students in the practical year in general and depending on different specialized veterinarian

Demographics:

- Year of birth

- Gender

- Previous training/courses

1. Has a preliminary meeting taken place in which learning objectives, expectations and framework conditions were discussed and defined?

1.1 Yes: How did this preliminary meeting go?

1.2 No: Looking back, would you have wished for such a preliminary discussion?

2. Was specialization of the veterinarians important when choosing the internship? Please explain.

2.1 Yes: Why was it important to you?

2.2 No: Why was it not particulary important to you?

3. Please describe whether and to what extent you felt differently cared for by specialized or non-specialized veterinarians.

4. Were you able to carry out many practical activities?

5. Who instructed and had you performed the practical skills?

6. How would you rate the quality of the instructions for performing the practical activities?

7. Is there a difference between guidance from a veterinarian with or without specialization?

8. How was your learning progress checked during the internship (e.g. logbook)?

9. Looking back, would you place more emphasis on the specialization of veterinarians when choosing an internship? Please explain.

10. Is there anything else you would like to add on the subject of “Supervision of students during the practical year”?

**4 Interview guidelines veterinarians**

Topic: Evaluation of the supervision and clinical training of students during the practical year as well as the influence of specializations of the supervising veterinarians

Aim: Recording subjective experiences in relation to the supervision of students during the practical year

Demographics:

- Year of birth

- Gender

- Specialization

- Practice or clinic, animal species

- Number of employed veterinarians

1. Who is responsible for supervising students during their practical year at the practice/clinic where you work?

1.1 Are you involved in the supervision of students in their practical year?

2. Why are the people you mentioned involved in supervising the students?

3. Are employees of the practice/clinic trained in dealing with interns?

3.1 Yes: How the employees are trained?

3.2 No: How is it checked that employees are confident in dealing with interns

4. How would you rate the theoretical knowledge, practical skills and communication skills that students bring with them from their study period?

5. Which skills are most important to you and why?

6. Is there a guideline for checking student´s learning progress?

6.1 Yes: How is this guide structured?

6.2 No: Is there any other way to check the learning success of the students?

7. What expectations do you have of students?

7.1 Specialization: Do you have different expectations of student education due to you specialization?

7.2 Without specialization: Do you think that specialization influences student´s educational expectations?

8. Is there anything else you would like to add on the subject of “Supervision of students during the practical year”?
